# The Origins and Functions of De Novo Genes: Against All Odds?

**DOI:** 10.1007/s00239-022-10055-3

**Published:** 2022-04-22

**Authors:** Caroline M. Weisman

**Affiliations:** Lewis-Sigler Institute for Integrative Genomics, Princeton University, Princeton, NJ USA

**Keywords:** De novo genes, Lineage-specific genes, Taxonomically-restricted genes, Evolutionary novelty, Genetic novelty, Gene birth

## Abstract

“De novo” genes evolve from previously non-genic DNA. This strikes many of us as remarkable, because it seems extraordinarily unlikely that random sequence would produce a functional gene. How is this possible? In this two-part review, I first summarize what is known about the origins and molecular functions of the small number of de novo genes for which such information is available. I then speculate on what these examples may tell us about how de novo genes manage to emerge despite what seem like enormous opposing odds.

## Jacob’s Conundrum: The Apparent Implausibility of De Novo Genes

As proudly noted in the introduction of many papers on the subject, the birth of new genes out of non-genic sequence—the “de novo” origin of genes—was once regarded to be essentially impossible. An excerpt from Francois Jacob’s famous *“Evolution and Tinkering”* has become the compulsory citation for this view: as he put it, “the probability that a functional protein would appear de novo by random association of amino acids is practically zero” (Jacob 1977).

Since then, clear examples of de novo genes have defiantly demonstrated that this probability is *not* zero. Jacob’s claim was wrong. Nonetheless*,* it remains compelling*,* voicing an intuition that most of us still share: that a protein made of largely unselected sequence is *enormously* unlikely to do something useful for the cell.

I propose the following argument in support of Jacob’s claim that de novo gene birth is prohibitively unlikely. Though he did not put it this way, I think it captures what many of us find compelling about it.

**Table:** 

**Premise 1 (“Sparsity”)**: A very small fraction of all possible sequences would produce a biological effect beneficial to the organism
**Premise 2 (“Fair play”)**: Non-genic sequences are a random and unbiased sample of all possible sequences
**Premise 3 (“Limited trials”)**: The number of non-genic sequences assessed for biological effects during evolution is modest
**Conclusion**: A gene evolving from non-genic sequence is very unlikely

Faced simultaneously with this seemingly compelling argument and with the reality of de novo gene birth, we have a conundrum. Why *is* de novo gene birth not so unlikely as to be impossible? Where has this argument gone wrong?

There are at least three (not mutually exclusive) possibilities, corresponding to violations of each of the three premises.

**One** An appreciable fraction of all possible sequences would produce a biological effect beneficial to the organism.

**Two** De novo genes emerge from sequences that are, compared to a truly random sample, somehow enriched for beneficial biological effects.

**Three** The *number* of sequences tested by evolution is sufficiently high that it successfully samples the very small *fraction* of sequence space that has beneficial biological effects.

## A Case Study Approach to Understanding De Novo Gene Emergence

Here, I take a case study approach to understanding de novo genes. First, I summarize what is known about the fairly small number of de novo genes for which molecular details of their origins and biological roles are available. I then speculate: I highlight what strike me as common features among these cases and hypothesize about what these might tell us about how de novo genes solve Jacob’s conundrum of implausibility. Readers uninterested in the detailed case studies may freely read only the speculation portion, or vice versa.

Other data—like experiments that directly query the properties of random sequences—bear on these questions; why opt for a case study approach? First, taken alone, an assembled set of de novo gene case studies should be independently informative; this aspect of the review can be considered independently of the rest. Second, this approach adheres to the simple-minded theory that it is essential to look to these genes themselves to understand how they came to be. More indirect inferences, though they have other advantages, may not be representative of processes and forces as they have played out in nature. Third, a case study approach has, to my knowledge, not been performed before, and different approaches to the same question are always desirable, increasing confidence in shared conclusions and prompting closer consideration of unique ones. To that end, while a comprehensive review of other relevant work is well beyond my scope here, I do not restrict myself to case studies, and so include highlights from these other approaches, especially those that present interesting agreement or disagreement with conclusions from the case studies.

## Criteria for Inclusion of a Gene in this Review

There is substantial work identifying and characterizing de novo genes, already well summarized elsewhere (McLysaght and Guerzoni 2015; McLysaght and Hurst 2016; Oss and Carvunis 2019). For our purposes, much suffers from two limitations. First, there has been some lack of consensus about what level of evidence is desirable to conclude that a gene has a de novo origin, and is not, for example, a conserved gene whose homologs in other species have not been successfully detected (McLysaght and Guerzoni 2015; McLysaght and Hurst 2016; Vakirlis et al. 2020a; Weisman et al. 2020). As a result, much work has used methods prone to technical artifacts that include many (Vakirlis et al. 2020a)—potentially a large majority (Weisman et al. 2020)—false positives: genes that are not actually de novo. Conclusions reached from these potentially polluted samples would be fraught. Second, the biological effects of purported de novo genes are rarely probed directly; more often, accessible but imprecise proxies (e.g., expression pattern) are used, which are not strongly informative for the molecular and cellular understanding that we seek.

Here, I consider only genes whose de novo status is strongly supported and whose biological effects have been directly characterized. I have applied the following criteria.

First, I require positive evidence of the gene’s absence from outgroup species. For RNA genes, there must be evidence that the orthologous sequence is not transcribed, or that it produces a substantially different transcript, in outgroups. For protein-coding genes, there must be evidence that the orthologous sequence is not translated, or that that the ORF is substantially different, in outgroups. Note that the failure of, e.g., BLAST to detect homologs in outgroups, a common methodology, does not constitute such evidence.

Second, I require at least two outgroups for which the above is true. This is the minimum number required to make de novo gene gain likelier than the alternative of gene loss in outgroups, assuming (generously) that these events are equally likely.

Finally, I require data suggesting that the gene has a biological effect, in the form of an observable phenotype when it is knocked out or down. For protein-coding genes, there must be some evidence that this phenotype is due to the novel protein rather than the transcript. (As others have noted, the word “function,” especially for de novo genes, is fraught (Keeling 2019); when I use it here, it is as shorthand for this criterion of “producing a biological effect,” and does not imply other frequently associated concepts like having been evolutionarily selected.)

Many will regard these criteria as conservative. They are still imperfect. For example, an outgroup sequence inferred to lack a sufficiently similar open reading frame may yet be initiated by a noncanonical start (Chen et al. 2020). Ideally, the absence of translation or phenotypic consequence in outgroups would be directly demonstrated. But these and other more rigorous tests are difficult and rarely performed.

Such conservatism comes with costs. Not many genes meet these criteria, producing the limitations of small sample sizes. Those that do may not be representative. For example, many genes included here are from vertebrates or yeast, or are involved in cancer; I suspect that this at least partially reflects ascertainment bias resulting from these systems being particularly tractable and well studied. Nonetheless, as there are already comprehensive reviews taking less conservative approaches (Khalturin et al. 2009; Tautz and Domazet-Lošo 2011; McLysaght and Hurst 2016; Oss and Carvunis 2019), this strikes me as a worthwhile enterprise.

## Case Studies: Origins and Functions of Known De Novo Genes

### Protein-Coding Genes

#### Northern Gadid *AFGP*

The antifreeze glycoprotein AFGP is essential to the ability of Arctic codfish (gadids) to survive the cold temperatures of their environment: it is secreted into the blood, where it inhibits lethal formation of ice crystals (Cheng 1998). AFGP evolved de novo in the gadid lineage about 3 million years ago, presumably in response to the Pliocene glaciation event (Zhuang et al. 2019).

AFGP consists of a large and variable (20–500) number of Thr-(Ala/Pro)-Ala repeats, preceded by a signal peptide that enables secretion, and by a small glutamine-rich propeptide that is removed post-translationally (Zhuang et al. 2019). The threonine in each repeat is glycosylated with an O-linked N-acetyl-D-galactosamine (Cheng 1998), presumably via the standard O-glycosylation pathway in the Golgi, to which it is likely targeted by standard amino acid sequence determinants (Gill et al. 2011).

Structural work suggests that AFGP exists in an ensemble of conformations, comprised mostly of random coils, polyproline-II helices, and alpha helices (Giubertoni et al. 2019). Compounds that inhibited or enhanced AFGP’s antifreeze effects did so without significantly altering its structure, implying that, consistent with previous results (Devries 1971), its antifreeze activity is due largely to ice crystals interacting with the hydroxyl groups on the galactosamines, rather than with the peptide backbone or side chains themselves.

There have been many independent origins of antifreeze proteins (Cheng 1998). Even among these, AFGPs in polar fish are an example of particularly precise evolutionary convergence. A separate lineage, Antarctic notothenioid fish, has independently evolved an antifreeze glycoprotein with essentially *the same sequence* as the arctic gadid AFGP. This notothenioid protein is, however, *not* de novo in origin: its 5′ end, including the secretory signal, and part of its 3′UTR—though not its repetitive coding sequence—are derived from an ancestral protease (Chen et al. 1997).

#### *Saccharomyces cerevisiae* MDF1

*MDF1,* one of the first experimentally characterized de novo genes (Li et al. 2010), is a 153 amino acid protein found only in the budding yeast *S. cerevisiae,* in which it likely originated de novo within the last few million years. Knockout and rescue experiments show that MDF1 increases vegetative (aka asexual) growth in haploid cells (Li et al. 2010) through at least two seemingly independent mechanisms.

First, knockout and rescue experiments show that MDF1 represses expression of genes in the mating pathway, whose basal expression reduces proliferation in *S. cerevisiae.* In diploids (which have already mated), the mating pathway is repressed by two transcription factors, MATalpha2 and MATa1, which dimerize to bind and repress promoters of mating pathway genes. In alpha-type haploids (one of two ‘mating types’ in *cerevisiae*), the mating pathway is not fully repressed because MATa1 is not expressed, leaving MATalpha2 without its necessary binding partner. MDF1, however, *is* expressed in alpha haploids. MDF1 binds to MATalpha2 and, in its presence, to the promoters of mating pathway genes, at the same positions as does the MATa1-MATalpha2 dimer in diploids. Moreover, MDF1 is computationally predicted to have a structure fairly similar to that of MATa1, sharing the presence and relative orientation of three essential alpha helices. Taken together, these and other experiments suggest that MDF1 acts as an alternative context stand-in for MATa1, producing diploid-like repression of mating pathway genes in haploid cells (although, as alpha haploids *can* still mate, not as effectively) (Li et al. 2010).

In a potentially orthogonal mechanism, MDF1 has been proposed to increase vegetative growth by decreasing the time spent in “lag phase,” a characteristic time delay between when yeast are exposed to fresh fermentative media and when they begin exponential growth. It is believed that this delay in part reflects the time required to activate fermentation pathways and repress alternative respiration pathways. Overexpression of MDF1 decreases both the time spent in lag and the expression of genes involved in respiration. MDF1 also binds to the kinase SNF1, which activates respiration genes and represses fermentation genes. The data are consistent with the proposed hypothesis, but with the caveat that only overexpression was tested, with no supporting knockdown evidence (Li et al. 2014).

Like several other genes that follow, the regulation of MDF1 seems to have an intriguing property. It is antisense to and partially overlaps ADF1, a conserved gene. ADF1 overexpression negatively regulates MDF1 levels, and ADF1 was shown to bind at the MDF1 promoter. One explanation of these data is that MDF1 is regulated by the protein product of ADF1, the gene that it happens to lie beneath (Li et al. 2010).

#### *Saccharomyces cerevisiae* BSC4

BSC4 (to my knowledge, the first reported gene meeting the criteria used here) encodes a 132 amino acid protein specific to *S. cerevisiae* (Cai et al. 2008). The homologous locus is transcribed, but lacks an open reading frame encoding a significantly similar protein, in four closely related yeasts. In a ribosome profiling dataset of the closest outgroup *S. paradoxus* (McManus et al. 2014), I find extremely low but nonzero expression (~ 0.2 RPKM), suggestive of ancestral ‘leaky’ translation.

Despite its early identification, little is known about BSC4’s biological role. It undergoes stop codon readthrough and is expressed in standard media and upregulated in stationary phase; and a deletion of the locus is lethal when combined with deletions of either of two conserved genes, RPN4 and DUN1 (Pan et al. 2006; Li et al. 2010). Both of these genes have reported roles in DNA repair (Cai et al. 2008), but also have others. It is not clear if BSC4, and not some other feature captured by the deletion, is responsible for this phenotype.

BSC4 was also to my knowledge the first de novo gene subjected to experimental structural characterization (Bungard et al. 2017). It was found to be rich in beta sheet secondary structure and to adopt a likely “molten globule” structure: it has a hydrophobic core with buried residues, and so is not totally disordered, but does not have a single, stable fold. It also was found in refolding experiments to form oligomers of sizes up to hexamers (Bungard et al. 2017).

#### *Homo sapiens* PBOV1

PBOV1 is a 135 amino acid human protein that lies entirely within an intron of the conserved gene BIG3, on the opposite strand. It likely originated de novo in humans or possibly hominid primates (An et al. 2000).

PBOV1 was first identified as being overexpressed in prostate and breast cancer (An et al. 2000). In prostate cancer cells, PBOV1 knockdown decreases cell proliferation and anchorage-independent growth. PBOV1 knockdown also resulted in reduced progression through the G1-S checkpoint, decreased levels of G1-S transition inhibitors (p21 and p27), and increased levels of G1-S activators (cyclin d1 and phosphorylated Rb) (Pan et al. 2016), suggesting that the proliferation phenotype is achieved through affecting cell cycle regulation.

In hepatocellular carcinoma lines (Guo et al. 2018), PBOV1 knockdown also reduced proliferation, migration, survival, and metastatic potential (measured by cell penetration of a basement membrane). Subsequent experiments have suggested several potential mechanisms for these effects. In the same study, PBOV1 knockdown decreased progression through the G1-S checkpoint and cyclin d1 levels, again suggestive of PBOV1 affecting cell cycle regulation.

PBOV1 was also shown to interact directly with beta-catenin, and to inhibit it from being phosphorylated by GSK3B, preventing its subsequent degradation. This effect was dose-dependent, suggesting competitive inhibition. PBOV1 knockdown also reduced the expression of pluripotency factors Oct4, Nanog and c-Myc, and decreased the levels of cleaved caspase-3 (a marker of apoptosis) (Guo et al. 2018). Mechanisms for these effects were not explored.

PBOV1 knockdown also caused a suite of expression changes in wnt/beta-catenin regulator HIF1A, downstream targets of beta-catenin, epithelial markers (alpha-cadherin and E-cadherin), and mesenchymal markers (N-cadherin and vimentin) that are characteristic of those found in the “epithelial-to-mesenchymal transition” (EMT). The EMT is a developmental program, important in embryogenesis, in which epithelial cells lose cellular adhesion and gain motility, allowing them to migrate. The EMT has been found to occur in many cancer types: it is hypothesized that reactivation of this embryonic program allows epithelial cells to migrate and form metastases (Brabletz et al. 2018). Several other genes below also seem to induce the EMT in various cancer types.

#### *Homo sapiens* NYCM

NCYM is a 109 amino acid human protein named for its location: it is antisense to and partially overlaps the well-known oncogene MYCN (the two coding regions do not overlap). NCYM likely emerged either uniquely in humans or prior to the split with chimpanzees. It is arguably the best experimentally characterized of all de novo genes. Here I include only major highlights; a thorough review is available elsewhere (Suenaga et al. 2020).

MYCN is frequently amplified in several cancer types, most notably in neuroblastoma (Kohl et al. 1983), where it is well known to enhance cell proliferation and survival (Huang and Weiss 2013); NCYM is co-amplified with it as a result of the genomic architecture of the two genes (Kang et al. 2006), and it too is regarded as contributing to oncogenesis. In a study in neuroblastoma lines, NCYM knockdown reduced tumor sphere formation and symmetric cell division (two characteristics of undifferentiated cells, taken to be indicative of self-renewal potential contributing to oncogenesis in this stem cell-like cancer type) (Kaneko et al. 2015). In a different neuroblastoma line study, NCYM knockdown reduced apoptosis (Shoji et al. 2015). NCYM knockdown also reduced tumor sphere formation and tumor size in bladder cancer lines (Zhu et al. 2018). And a somewhat unphysiological result comes from a mouse model of neuroblastoma, created by expressing high levels of human MYCN in neuroectoderm (Weiss et al. 1997): here, additional expression of NYCM did not increase number of tumors, but did increase the number of distant metastases (Suenaga 2014).

NYCM is reported to have effects on several other genes involved in cancer, presenting possible mechanisms for these oncogenic phenotypes. In MYCN-amplified neuroblastoma lines, NCYM knockdown decreased levels of MYCN protein in a proteasome-dependent manner (Suenaga 2014). NYCM was found to co-immunoprecipitate with MYCN and kinase GSK3B, which natively phosphorylates MYCN to target it to the proteasome for degradation (Sjostrom et al. 2005), in both neuroblastoma (Suenaga 2014) and bladder cancer lines (Zhu et al. 2018). In vitro, NYCM inhibited this phosphorylation, but was not itself phosphorylated, so likely not by competitive inhibition (Suenaga 2014). In the mouse neuroblastoma model, NCYM overexpression increased beta-catenin levels (Suenaga 2014), and in bladder cancer lines, NCYM knockdown decreased beta-catenin levels and increased E-vimentin levels; these changes are characteristic of the EMT, and are consistent with an underlying mechanism in which NCYM inhibits GSK3B (Suenaga 2014) to stabilize its substrates, which include beta-catenein and E-vimentin. Another interaction partner was suggested by in vitro experiments in which NCYM increased cleavage of MYCN and interacted with the responsible protease (Shoji et al. 2015), whose product, “Myc-nick,” has other oncogenic effects (Conacci-Sorrell et al. 2014). And, in neuroblastoma lines, NCYM knockdown decreased both Oct4 expression and MYCN binding to a known “E-box” enhancer element that drives Oct4 expression (Kaneko et al. 2015), suggesting that it regulates Oct4 via MYCN. An increase in Oct4 was not observed in the mouse neuroblastoma model expressing NYCM, consistent with the observation that the E-box element to which MYCN binds is not conserved (Kaneko et al. 2015).

Complex regulatory interactions between NYCM and MCYN have been reported. NYCM expression is regulated by the MYCN protein via an E-box enhancer (a short palindromic element) within the NYCM gene body (Suenaga 2014), which is the same one used by MYCN to positively regulate its own expression (Suenaga et al. 2009). The MYCN protein is in turn stabilized by NYCM in a feed-forward regulatory loop (Kaneko et al. 2015).

Recent work on the secondary structure of NCYM reports that it contains alpha helices and beta strands, and forms a mixture of monomers, which localize to the nucleus, and oligomers ranging in size up to tetramers, which localize to the cytoplasm (Matsuo et al. 2021). The difference in quaternary structure with subcellular location may underlie the quite different effects that NYCM has been reported to have.

It is generally believed that both the NCYM protein and the NCYM RNA have biological roles. Knockdown experiments do not distinguish between these, but a role for the protein per se seems likely given in vitro and co-immunoprecipitation results consistent with knockdown phenotypes. The most compelling support for a transcript-specific role comes from one experiment where overexpression of the NCYM transcript increased MYCN expression, even when the protein ORF was disrupted (Zhao et al. 2016). Other data may be characterized as suggestive of transcript-specific roles. In neuroblastoma cell lines, where NCYM transcript knockdown altered levels and isoforms of MYCN RNA (Vadie et al. 2015), the transcript itself was shown to localize to the MYCN locus. In different cell lines, where CTCF was found to positively regulate MYCN levels, the NYCM transcript coimmunoprecipitated with CTCF, and NYCM knockdown reduced CTCF-mediated increase of MYCN levels. Overall, it seems likely that both molecules produce some effect(s), and the in vitro assays above give reasonable support for a few in particular that belong to the protein, but overall, the attribution of specific roles to each molecule remains ambiguous.

The NCYM peptide emerged in a subset of the primate lineage; the locus is conserved in mammals (Suenaga 2014) and is expressed in at least one baboon species (per transcriptome data available at NCBI: Bioproject PRJNA167997). This is consistent with an ancestrally noncoding RNA giving rise to a de novo protein in primates. Assigning roles specifically to the clearly de novo *protein* is especially important given that the RNA may be conserved.

#### *Homo sapiens* MYEOV

MYEOV is a 313-amino acid protein that emerged in the human lineage (Chen 2015; Papamichos et al. 2015), first identified in a screen for the ability of DNA from gastric carcinoma to induce tumor formation (Janssen et al. 2000). Subsequent studies have explored its oncogenic roles in a variety of cancer types.

In colorectal cancer lines, MYEOV knockdown reduced cell proliferation (Lawlor et al. 2010) and invasion (Moss et al. 2006; Lawlor et al. 2010), and its transcript levels were responsive to prostaglandin E2 (Lawlor et al. 2010). Particularly detailed data come from a study in pancreatic cancer cells (Liang et al. 2020). In two cell lines, MYEOV knockdown reduced cell migration, invasion, and proliferation in vitro and reduced tumor weight and liver metastasis formation in vivo. In these experiments, multiple assays in both lines found that MYEOV binds to the transcription factor SOX9 and colocalizes with it in the nucleus. MYEOV knockdown decreased transcript levels of the gene HES1, and also decreased SOX9 binding at the HES1 enhancer. MYEOV itself bound the same HES1 enhancer sequence; knockdown of SOX9 reduced this binding. Finally, knockdown of either HES1 or SOX9 abolished the ability of MYEOV overexpression to increase cell migration and invasiveness. Collectively, these results suggest that MYEOV and SOX9 dimerize to drive expression of HES1, which effects downstream oncogenic phenotypes.

Existing knowledge of HES1 and SOX9 supports this as a plausible mechanism. HES1 is a known target of notch signaling that has been shown to increase pancreatic cancer cell migration and invasiveness (Abel 2014). SOX9 has been shown to help maintain the pool of pluripotent progenitor cells in the pancreas (Seymour et al. 2007), partly through regulating HES1 (Belo et al. 2013).

SOX9’s requirement of MYEOV to drive HES1 expression is also not without precedent. A study in breast cancer cells found that SOX9 homodimerizes at the same HES1 enhancer as in the MYEOV study to drive transcription (Müller et al. 2010). In its non-oncogenic roles, SOX9 acts in some contexts as a monomer and in others as a homodimer (Bernard et al. 2003), depending strongly on its concentration (Sock et al. 2003). A speculative take is that in pancreatic cancer, low concentrations of SOX9 that would ordinarily fail to produce the dimers necessary for driving HES1 expression are overcome by sufficient levels of MYEOV, which acts as a stand-in.

The MYEOV transcript itself also seems to have effects. Although the ORF is absent in outgroups, the locus is present in most primates (Papamichos et al. 2015) and transcribed in chimpanzee (Chen 2015). In lung cancer lines (Fang et al. 2019), the transcript was expressed, but no protein was detected. Consistent with a previous report (Almeida et al. 2006), removing the 5′UTR resulted in translation, possibly due to alleviation of interference from several small upstream open reading frames (uORFs) within it. Differences in regulation of these uORFs in different cell types may account for the protein being detected in pancreatic cancer cells and not here.

In these lung cancer lines, MYEOV bound miRNAs targeting two components of the TGF-B/SMAD pathway, USP15 and TGBR2. In vivo, MYEOV knockdown reduced tumor invasion and metastasis, reduced expression signatures characteristic of the EMT, and reduced TGF-B signaling; constitutive activation of TGF-B signaling rescued these phenotypes. MYEOV overexpression produced effects opposite to those observed in knockdown and required the predicted binding site of the miRNAs, but not the ORF, to be intact (Fang et al. 2019). Between in vitro assays involving the MYEOV protein itself and these experiments, there is good evidence that both the transcript and the protein have roles and that the protein per se acts as a transcription factor.

## RNA Genes

### *Homo sapiens* ELFN1-AS1

ELFN1-AS1 is an RNA gene unique to humans, lying entirely within an intron of the conserved gene ELFN1, and encoded on the opposite strand (Polev et al. 2014). It is expressed in a variety of tumor types, but only at low levels in normal tissue (Polev et al. 2014).

Knockdown experiments in different cancer cell lines, including colon (Dong et al. 2019; Du et al. 2021), esophageal (Zhang et al. 2020), and ovarian cancer (Jie et al. 2020), show that ELFN1-AS1 promotes oncogenesis by increasing cell proliferation and migration. In one colon cancer study (Dong et al. 2019), ELFN-AS1 knockdown also caused expression changes characteristic of repression of the EMT.

One mechanism for these oncogenic effects is thought to be via action as an microRNA sponge. Knockdown experiments have identified multiple miRNAs and corresponding target genes: TRIM44 (Dong et al. 2019) and SATB1 (Du et al. 2021) in colon cancer, GFTP1 (Zhang et al. 2020) in esophageal cancer, and CLDN4 (Jie et al. 2020) in ovarian cancer. Though only some of these genes had been previously known as involved in oncogenesis (Wei et al. 2019), in all cases, the effects of ELFN-AS1 knockdown were at least partially rescued by inhibition of the suspected miRNAs and by downregulation of their suspected targets (Dong et al. 2019; Zhang et al. 2020; Jie et al. 2020; Du et al. 2021). Not yet understood are the mechanisms downstream of these targets; whether these mechanisms are related; and if they are simultaneously active in the same cell types, or are instead context-dependent.

### *Mus musculus* Poldi

*Poldi* is a noncoding RNA present in several *Mus* species including *M. musculus*, and likely emerged in the lineage around 3 million years ago. In *M. musculus*, it is expressed specifically in the postmeiotic round spermatids of the seminiferous tubules. Mice with a deletion of the *Poldi* locus exhibited somewhat decreased sperm motility and testis weight (Heinen et al. 2009), although the possibility another feature within the relatively large region underlying this effect was not tested, e.g., by a rescue experiment, and mechanisms underlying the observed effect remain unknown.

## Speculation: How do de novo Genes Beat the Odds?

I now highlight apparent commonalities among these examples and consider what they may tell us about how de novo genes avoid Jacob’s conundrum of improbability, supplementing where appropriate by insights from other literature. This section is speculative: there are too few examples to be sure that apparent similarities are not due to chance, and many proposed causes are highly conjectural.

### Pervasive Transcription and Translation Offer Many Opportunities for De Novo Birth

I would be remiss not to mention a solution to the conundrum suggested not by these case studies but by evidence from technologies like RNA-seq and ribosome profiling: that the number of “trials” for de novo birth is much greater than previously believed.

The raw material for these trials is the large amount of noncoding DNA present in most genomes, especially those of “higher” eukaryotes. Recent work has revealed that surprisingly large amount of this sequence, despite neither having apparent bioactivity nor being under detectable selective constraint, is subject to low levels of “pervasive” and “promiscuous” transcription and translation (Clark 2011; Ingolia et al. 2014). Comparative work also suggests that which particular sequences that are expressed at a given time changes quickly over evolution (Neme and Tautz 2016; Ruiz-Orera et al. 2018; Durand et al. 2019). These suggest that a vast number of sequences are tested during evolution (Wilson and Masel 2011; Carvunis et al. 2012), violating our “limited trials” premise.

### Basic Structural Properties are Easy to Come By

We might imagine that it is unlikely for a random sequence to have defined structural features that we associate with established proteins: alpha helices, beta sheets, globularity, and so on. But many of these genes *do* seem to have these features.

This notion is consistent with work computationally and experimentally characterizing the structural and biophysical properties of random sequences libraries, which has shown that random sequences are surprisingly rich in alpha helices and beta sheets (on average, ~ 10–20% content of each) (Tretyachenko et al. 2017; Heames et al. 2022) and transmembrane domains (~ 40% of sequences with at least one) (Vakirlis et al. 2020b).

Related work has considered intergenic open reading frames (ORFs), which are clear candidates for the raw material from which de novo genes most directly arise (Carvunis et al. 2012; Vakirlis et al. 2020b). Though the vast majority of these are not known to have biological effects, their sequences may still not be random, potentially shaped by the indirect action of selection or by other nonrandom processes. These intergenic ORFs have been found to have several computational measures associated with high foldability (Papadopoulos et al. 2021) and to be even more enriched for transmembrane domains than their composition-matched random counterparts (Vakirlis et al. 2020b).

So basic structural elements may be more common in sequence space than we expect, softly violating our sparsity premise (while disordered proteins show that defined structure is not *essential* for bioactivity, it may facilitate at least some types of effects). There may also be differences between the structural properties of non-genic DNA and those of truly random sequences: a violation of fair play.

The finding that intergenic ORFs are enriched in transmembrane sequences synergizes with recent random sequence studies. In *E. coli*, peptides conferring resistance to two different antibiotics found in screens of random sequence libraries were shown to do so via their position in the cell membrane (Knopp et al. 2019, 2021). A third study in *E. coli* found that several peptides from a random library induce upregulation of genes in the phage shock pathway (Bhave and Tautz 2021), which is activated in response to inner membrane permeability (Flores-Kim and Darwin 2016); notably, though, these peptides *decreased* fitness.

Despite these suggestions that membrane localization may be highly accessible to de novo genes, no examples here have been shown to have this feature. Beyond our small sample size, this could be due to as-yet undiscovered properties of these genes. There may also be differences between natural evolution and experimental conditions in these studies: even within them, the reported fitness effects of transmembrane domains are sometimes positive but sometimes negative. Nonetheless, the “transmembrane-first” (Vakirlis et al. 2020b) model remains intriguing, and stands as to my knowledge the first proposed cell biological mechanism for de novo gene birth.

### Some Biological Effects Require Only Small Regions of Watson–Crick Complementarity and So are Common in Sequence Space

Two genes, MYEOV and ELFN1-AS1, alleviate existing miRNA suppression regulating established cellular pathways: they “sponge” them up, preventing them from effecting the intended suppression.

The sequence requirements to act as a miRNA sponge are minimal: an exact match of only 6–8 nucleotides is often sufficient complementarity for target binding (Bartel and MicroRNAs, 2009). Sequence space should be rife with them, violating our sparsity premise. This should also be true of other biological effects for which small numbers of complementary nucleotides are sufficient, including miRNAs themselves. Although they do not quite meet the criteria that I use here, existing reports are suggestive of widespread miRNA birth in a variety of taxa (Fahlgren 2007; Lu et al. 2008; Meunier et al. 2013).

### Overlap with a Conserved Gene Lowers the Barrier to Expression

A gene must not only encode a bioactive product, but have a context allowing that product to get to the right place at the right time. This presents many hurdles. The gene must lie in open chromatin; have regulatory elements that drive its expression; be exported from the nucleus and stable in the cytoplasm; and so on. The abundance of beneficial biological effects in sequence space depends on the abundance of elements conferring these properties.

Four genes here (NYCM, MDF1, PBOV1, and ELFN1-AS1) overlap with more conserved genes, consistent with other surveys of de novo genes identified using different methods (Knowles and McLysaght 2009; Murphy and McLysaght 2012). As noted previously (Murphy and McLysaght 2012), this feature likely at least in part reflects ascertainment bias: it is easier to demonstrate that a gene has emerged de novo if it overlaps a conserved “anchor gene.” But this may also be a genuine feature. Overlapping an existing gene jumps many hurdles to expression, co-opting many of these features that are already in place to drive expression of the conserved gene (Murphy and McLysaght 2012) and violating our fair play premise.

Moreover, two genes, NCYM and MDF1, seem to be transcriptionally regulated by the *protein products* of their overlapping genes*.* This seems an enormous coincidence: why should a protein return to act, *of all places*, so close to its native locus? There is a parsimonious explanation for NYCM. MYCN uses a regulatory sequence in its own autoregulation; because this falls conveniently within NYCM, it can drive expression of old and new genes alike. (This dual use may be enabled by this particular enhancer’s palindromic sequence, or may merely reflect what has been suggested to be an innate bidirectionality of promoters and enhancers in general (Xu et al. 2009; Jin et al. 2017).) Such autoregulation has not previously been noted for ADF1, but may merely not yet have been discovered, or may have been ancestrally present and since lost. So this striking regulatory interaction may be another failure of our fair play premise.

On the flip side, although many of these genes may have used this strategy to acquire expression, many others have not, suggesting that expression itself may not be quite so rare in sequence space as we imagine. Indeed, recent work has demonstrated a surprisingly high probability of a basal level of promoter activity) among random sequences: in *E. coli*, ~ 10%, with 60% one mutational step away) (Yona et al. 2018; Lagator, et al. 2022), and in yeast, 83% (Boer et al. 2020). A violation of the sparsity premise is also likely at play.

### Noncoding Function Lowers the Barrier to Coding Expression and Function

Two genes, MYEOV and NCYM, appear to encode both a functional protein and a functional RNA. In both cases, the protein emerged recently, but the locus itself is more deeply conserved and appears to be transcribed in outgroups; this suggests that the RNA came first, and the protein emerged within it later. This scenario has been previously proposed as a general mechanism for de novo gene birth (Wilson and Masel 2011; Reinhardt 2013; Chen 2015; Ruiz-Orera et al. 2018), and would have a clear benefit: the same violation of Jacob’s fair play premise as above.

A second benefit to this mechanism relates to the observation that non-genic ORFs with no identified function inside of transcripts are ‘promiscuously’ translated at low levels (Ingolia et al. 2014; Ruiz-Orera et al. 2018). This exposes them to the effects of selection. Although it seems too strong to say that *function* is selected for at this stage, selection for or against other properties that affect the cell when peptides are promiscuously translated may bias them toward areas of sequence space that happen, secondarily, to be enriched for function. To my knowledge the first proposal of such a mechanism, and the best-supported example, suggests that selection acts “preadaptively” on non-genic, promiscuously translated ORFs to reduce their propensity to aggregate and thereby harm the cell (Wilson et al. 2017; Kosinski et al. 2021). Selection resulting from promiscuous expression for this or other features could bias the sequence space from which de novo proteins emerge.

### New Proteins Inherit Older Noncoding Functions

For both MYEOV and NCYM, protein and the RNA—distinct molecules—are reported have very similar functions. For NCYM, they stabilize the same protein; for MYEOV, they activate the same pathway (Fang et al. 2019; Liang et al. 2020). As discussed, this apparent similarity may merely be imperfect experimental separation of the functions of the RNA and protein. If real, it is a striking coincidence. We might speculate that ‘inheriting’ the function of a host transcript somehow enables de novo gene birth.

How? One possibility: being encoded at the same locus causes the RNA and protein to share other features that themselves contribute to function. A protein and its transcript may share expression timing, cellular localization, and interaction partners (Berkovits and Mayr 2015). Born to be in the same place, at the same time, and in the same company as the RNA, it is perhaps not surprising that the protein evolves a similar job.

Another possibility: producing a new protein from an RNA locus reduces the RNA available for its function—its transcripts now sequestered by ribosomes, or its locus occupied by polymerases producing different isoforms—putting pressure on the new protein to compensate by performing a similar job.

Yet another: genes encoded at the same locus resist being separated by recombination, allowing the evolution of beneficial positive epistasis from their actions in the same pathway, like ‘supergenes’ thought to evolve for this purpose (Thompson and Jiggins 2014). That mice transgenic for both NCYM and MYCN develop distant metastases at a rate much higher than those transgenic for either gene alone evokes this possibility (Suenaga 2014).

In these ways or others, a protein born atop an existing RNA may violate our fair play premise as it more easily evolves not only *a* function, but is also predisposed toward the *particular* function performed by that RNA. For new genes as well as for new humans, parentage may play a large role in future life. But, also as with humans, parental influence may fade with time. New genes may go on to evolve new and different roles, or even to move out of their childhood homes and relocate within the genome. With time, these genes may lose obvious marks of their origins; the number born this way could be larger than is apparent.

### Sequence Space "attractors" Increase the Probability of Function

The de novo AFGP’s many Thr-Ala/Pro-Ala repeats are essential for its antifreeze action. Reconstruction of the evolutionary history of the locus shows that these repeats are the result of extensive duplication of a single ancestral sequence (Zhuang et al. 2019). The same is true of the independently evolved (but not strictly de novo) AFGP in notothenoid fish (Chen et al. 1997). This remarkable similarity suggests that duplicating the single repeat is a highly evolutionarily accessible path to antifreeze activity.

We have so far discussed the probability of gene birth as defined by a single point in sequence space. If that point has a biological useful effect, birth succeeds; if not, it fails. But a nascent gene *moves* from point to point as it mutates, transitioning with probabilities given by the mutation spectrum. This spectrum is not uniform, and so neither is the direction of its motion; it lands on some points more easily than others.

There are points in sequence space that are not functional, but that have a comparatively high probability of being mutated into functional points. I call these points “attractors.” An example: a single short sequence, which can easily be turned into many repeats by processes, like tandem duplication and unequal crossing over, that have comparatively high mutational probabilities. The probability of finding function in sequence space includes the fraction of sequence space composed of these attractors, weighted by their probability of attraction. A function per se may be rare, but if it has many attractors, it is easier to find: a violation of our sparsity premise.

Another example is miRNA sponges. In discussing them above, I omitted an important detail: to meaningfully deplete the cellular miRNA pool, a miRNA sponge generally needs multiple binding sites, making it substantially rarer in sequence space. But a single binding site, highly abundant, is again, through ease of duplication, an *attractor* to multiple. Sequence space should be rife with these attractors.

### Interactions are Easy to Come By

An intuitive picture of how new proteins come to interact with existing proteins and pathways is that they at first have none or very few, and acquire them slowly, as selection painstakingly crafts them. It is striking that the examples here paint a very different picture. Many young proteins already interact with many molecules in many pathways.

Screens of random sequence libraries have probed the abundance of interactions with other cellular molecules in sequence space. In vitro screens of random amino acid libraries have found successful binders of a variety of proteins and small molecules; a tiny sampling includes ATP (Keefe and Szostak 2001), *E. coli* lipopolysaccharide (Morales Betanzos et al. 2009), streptavidin (Wilson et al. 2001), SH3 domain (Sparks et al. 1994), calmodulin (Dedman et al. 1993), and BiP (Blond-Elguindi et al. 1993). The reported frequencies of these successful binders surely depends on methodological details (what binding affinity threshold was used; how many binders were isolated and sequenced), but generally range from 10^–6^ to 10^–12^. Modest on their own, these are rates for individual substrates, such that the abundance of binding *something in the cell* should be much higher, and the underlying in vitro methods, like affinity purification, may not capture all physiologically relevant interaction strengths or conditions. Whatever the cause, other experimental setups have yielded much higher rates. For example, in an in vivo screen, 4% of sequences had transcriptional activator activity sufficient to drive GFP expression (Erijman et al. 2020). And, strikingly, 40% of a random library was initially insoluble but became so in the presence of bacterial chaperone DnaK, suggesting that it successfully facilitated their folding (Heames et al. 2022).

Why might interactions be so common? The picture of protein binding as based on the highly precise, highly favorable coordination of a few specific residues may be misleading. “Fuzzy binding,” in which lower-affinity associations create a cloudlike, dynamic, and dispersed interaction interface, has been increasingly recognized as an important mode of binding (Fuxreiter 2018); for example, DnaK binds fuzzily to clients (Rosenzweig 2017), though it is perhaps best appreciated among disordered proteins, like some transcriptional activators (Brzovic et al. 2011). In leveraging many common and weak interactions rather than a few rare and very strong ones, fuzzy binding may require less sequence specificity than more canonical binding, violating the sparsity premise.

Another possibility: perhaps existing proteins have been selected *to interact*. Existing interactions may facilitate new ones, either via particular existing interaction sites or through more general sequence features (like fuzzy binding). This would predict that the more binding partners a protein has, the more easily it can acquire new ones, a rich-get-richer effect consistent with scale-free distributions often found in cellular networks (Barabási and Albert 1999; Barabasi and Oltvai 2004). Indeed, in a remarkable coincidence, NCYM and PBOV1 both associate with GSK3B, a kinase noted both for its unusually large number of substrates (Beurel et al. 2015) and as a hub bridging pathways (Domoto et al. 2016).

A provocative proposal: perhaps binding is sufficiently common that protein interactions evolve akin to synapse formation in the brain, with initially abundant and promiscuous connections pared back to retain only those proving useful. Whatever its origin, abundant binding in sequence space would violate our sparsity premise.

### New Proteins Adopt Old Roles in New Contexts

Two genes, MYEOV and MDF1, act as transcription factors—but not alone, and not in new places. They dimerize with conserved transcription factors that, in different conditions, have different binding partners. They then drive expression at the same promoters as those conserved complexes to turn on the same pathways. In other words, the de novo protein performs the same role as the original transcription factor, but in a different *condition,* from which it is absent.

A protein’s function depends not just on its sequence, but on the many other molecules with which it interacts in carrying out its job. Acquiring the context necessary for a useful function may be much of the battle. Instead of constructing these contextual features themselves—a binding partner, a locus to bind, a useful pathway to activate—a new protein can merely masquerade as an old one that has left its post vacant. The ability of new proteins to easily slip into pre-fashioned roles would violate our sparsity premise.

### New Proteins Reactivate Existing Pathways in New Contexts

It is striking how many of these genes have roles in cancer. I think this is likely at least in part the result of ascertainment bias. Compared to work in other systems, cancer research seems not to rely as heavily on evolutionary conservation to indicate functional importance, and so is likelier to undertake studies of de novo genes. (An important corollary of this bias is that these genes may also have roles in non-cancer tissues that have not yet been discovered.) But there may also be genuine enrichment of oncogenic functions among de novo genes.

These oncogenic functions work through activation of conserved pathways and programs. For example, an even more strikingly specific commonality is that many of these genes promote epithelial-to-mesenchymal transitions. As noted above, the EMT is considered by some to be a program primarily used in development which is then reactivated in cancer, allowing mature tissues to aberrantly migrate and metastasize (Brabletz et al. 2018). Others of these genes aberrantly activate other pathways used elsewhere in normal physiology, like TGF-B signaling.

It is hard to imagine a de novo gene inventing a new route to cancer out of whole cloth. But it is easier to imagine one flipping one cellular switch, with the downstream effect of *reactivating* an existing pathway that was waiting, intact but silent. Elements of cancer can be considered just this kind of switch-flipping: unregulated, aberrant deployment of programs, like growth, migration, or vascularization, that in normal contexts help to *build* the organism, now let loose to wreak havoc. New proteins may generally find it easy to flip all kinds of cellular switches; cancer may often be the result. As above, such reactivation coming with relative ease would violate our sparsity premise.

### The Cell Offers Many “freeloader functions” that Require Little More than Binding and are Abundant in Sequence Space

Certain kinds of functions are notably absent from these examples: those resulting from the action of the gene more or less in isolation, like enzymes or motors. Instead, these new genes work by interacting with existing cellular players, modulating or co-opting established functions. Moreover, the physical sophistication of these interactions seems low. They are not finely tuned, molecularly precise movements, adding or removing single chemical groups or effecting subtle allostery; they happen, or can be easily imagined to happen, by *mere binding* to interaction partners. De novo genes *bind* miRNAs, soaking them up; *bind* complexes of kinases and substrates, stabilizing and destabilizing; *bind*, or *are bound* by, glycosylation enzymes and export machinery, receiving additions that *bind* ice crystals.

I refer to functions with these properties—(a) that modulate existing functions by (b) mere binding—as “freeloader functions.” That all our de novo genes have freeloader functions suggests that they are common in sequence space. Why might this be?

The distribution of function in sequence space is defined only in a particular cellular environment. Some functions are comparatively ‘self-powered’ and less dependent on this environment, like those of many enzymes merely requiring the presence of substrate. Other functions are highly dependent on environment: any component of a signaling pathway is useless without the upstream inputs and the downstream effectors that it links. Most proteins are quite dependent, only achieving their functions when interaction partners, cofactors, local physiological contexts, and other curated aspects of their particular cellular niche are in place.

Inverting this thinking, each component of a cell offers its own repertoire of opportunities for functions that can be achieved by altering it*.* Suppressing or activating a signaling component will effect a function: a change in signaling, mediated by the rest of the pathway in which it acts. The huge number of existing proteins and pathways in the cell collectively offer a correspondingly huge number of such functions*.* The two sections just above—new proteins adopting old roles, and activating existing pathways—might be viewed as particular forms that freeloader functions can take.

Altering the behavior of existing components in this way does not seem too difficult. In many cases, it is achievable by *mere binding.* Bind to a kinase and you may competitively inhibit its native reaction, or stabilize it; to any transcription factor, and you may enhance, or reduce, its DNA binding; to any glycosylase, and you may become a substrate. The delicate and dynamic interactions driving the cell can easily be perturbed by things *sticking* to them, and outward ripples result in functional phenotypes. And mere binding itself does not seem so likely to be rare in sequence space. So, freeloader functions may be quite common.

When we imagine biological function, a very specific sort springs to mind: striking, self-powered examples, like molecular motors and enzymes. But these are exceptions, not the norm. In using them as our yardstick to estimate how likely function is to emerge de novo, we forget that genes need not mostly act under their own steam. I propose that freeloading makes function vastly more common in sequence space than we have imagined, severely violating our sparsity premise.

## Looking Forward

Here, I have reviewed what is known about the origins and functions of the few de novo genes that I consider to be of high confidence and for which such information is available. I have also suggested themes among these examples, and speculated about what they mean for the question with which I opened: how do de novo genes solve Jacob’s conundrum of apparent implausibility? I emphasize again that this latter piece is speculation, its main aim being to draw attention to the question and to seed a few hypotheses for future work.

Insights will undoubtedly come from continuing efforts to experimentally characterize the properties of random amino acid sequences. Exciting highlights from recent work include in vivo searches of random libraries for particular biological roles (Knopp et al. 2019; Knopp 2021), biophysical characterizations of random sequences (Tretyachenko et al. 2017; Heames, et al. 2022), and assays of random libraries for more organismal-scale functions, like fitness consequences in an *E. coli* competition assays (Neme et al. 2017; Bhave and Tautz 2021; Fajardo and Tautz 2021) (though see (Weisman and Eddy 2017) and (Knopp and Andersson 2018) for serious caveats) and a variety of “visually conspicuous” phenotypes, including altered flowering time and loss of stamens, in *A. thaliana* (Bao et al. 2017).

Equally if not more important will be continuing efforts to identify *bona fide* de novo genes and experimentally characterize their origins and functions. Both planks in this enterprise are essential. For example, a spate of recent work has beautifully and convincingly characterized the molecular roles of genes that, lacking the strong evidence represented by the criteria that I use here, can only be called *putatively* de novo (Lange et al. 2021; Rivard 2021); similar efforts applied to genes whose de novo status is more unambiguous would be hugely informative. This approach and random sequence studies have complementary strengths. The former is arguably a more direct line to nature, not dependent on our ability to infer the conditions and beneficial features that hold in natural evolution; the latter, not beholden to the small number of routes that evolution has taken in fact, has the advantage of sheer power. It is my feeling, also noted by others (Eicholt et al. 2022), that experimental characterization of de novo genes lags behind other approaches; more focus here strikes me as essential.

In closing, I propose to reconsider a hypothesis that has prompted much of the interest in de novo genes: that, being *genetically* novel, they are likely to drive the evolution of *functional* novelty (Khalturin et al. 2009; McLysaght and Guerzoni 2015; Andersson et al. 2015). This is not particularly well supported by my analysis here: most of these genes do not do much that seems novel. The antifreeze AFGP is perhaps the exception—but since in another lineage essentially the *same sequence* evolved successfully from an existing gene, de novo birth seems to have no special claim to this new function. If our examples are representative, enriched as they are for freeloader functions, reprisals of existing roles, or reactivation of existing pathways, one might conclude, ironically, that, at least at the molecular level, de novo genes represent the *opposite* of novelty. We might now pause to reevaluate the widespread assumption that new genes are the best candidates for what lies beneath new features and should take care not to bias our efforts by assuming that this must be so. Even in inventing, evolution may yet be a tinkerer.
